# Risk factors for urinary infection after retrograde upper urinary lithotripsy: Implication for nursing

**DOI:** 10.1097/MD.0000000000026172

**Published:** 2021-08-06

**Authors:** Chuan Peng, Zhaozhao Chen, Jun Xu

**Affiliations:** Department of Urology, Wuhan Central Hospital, Wuhan, Hubei, China.

**Keywords:** care, retrograde upper urinary lithotripsy, stone, urinary infection, urology

## Abstract

There are needs to evaluate the risk factors for urinary infection after retrograde upper urinary lithotripsy, to provide insights into the management and nursing care of patients with retrograde upper urinary lithotripsy.

Patients who received retrograde upper urinary lithotripsy with a Foley 20 urinary tube insertion from June 1, 2019 to December 31, 2020 in our hospital were selected. Patients were grouped urinary infection and no infection group according to the culture results of urine, and the clinical data of the 2 groups of patients were collected and compared. Single factor and logistic regression analysis were used to analyze the risk factors of urinary tract infection after retrograde upper urinary lithotripsy.

Four hundred ten patients with retrograde upper urinary lithotripsy were included, of whom 62 patients had the urinary tract infection, the incidence of urinary tract infection was 15.12%. There were significant differences in the gender, age, diabetes, stone diameter, duration of urinary tube insertion and duration of surgery between infection and no-infection group (all *P* < .05). The Escherichia coli (62.90%) was the most commonly seen bacterial in patients with urinary tract infection. Female (odds ratio [OR]: 1.602, 95% confidence interval 95% [CI]: 1.132∼2.472), age >50 years (OR: 2.247, 95% CI: 1.346∼3.244), diabetes (OR: 2.228, 95% CI: 1.033∼3.451), stone diameter ≥2 cm (OR: 2.152, 95% CI: 1.395∼3.099), duration of urinary tube insertion ≥3 days (OR: 1.942, 95% CI:1.158∼2.632), duration of surgery ≥90 minutes (OR: 2.128, 95% CI: 1.104∼3.846) were the independent risk factors for the postoperative urinary tract infection in patients with retrograde upper urinary lithotripsy (all *P* < .05).

The incidence of urinary tract infection in patients undergoing retrograde upper urinary lithotripsy was high, counteractive measures targeted on those risk factors are needed to prevent and reduce the postoperative urinary infection in clinical settings.

## 1 Introduction

Urolithiasis is a common disease in the department of urology. The population prevalence is about 1% to 5%, and the recurrence rate after treatment is also very high.^[1]^ The recurrence rate of urolithiasis in 10 years after surgery is about 50%.^[2]^ In recent years, various intracavitary techniques have made great progress, including retrograde upper urinary lithotripsy and percutaneous nephrolithotomy.^[3]^ At present, more than 90% of urolithiasis cases do not require traditional open urinary tract stone removal.^[4]^ However, with the extensive development of intracavitary lithotripsy techniques, various postoperative complications have also followed. Among them, the incidence of postoperative urinary tract infection is higher, the incidence is 10.13% to 30.08%, which may develop into urinary infection related shock, and severe cases may even lead to death.^[5,6]^

How to reduce or even avoid the incidence of infection during the perioperative period in the treatment of urolithiasis by endourological surgery has been an issue of great concern to urologists in recent years. It has been reported at home and abroad that the use of antibiotics before surgery and the reduction of operative lithotripsy time during surgery can all reduce the rate of urinary tract infection and acute septic shock after surgery to a certain extent,^[7]^ but a considerable proportion of patients still cannot avoid severe urinary tract infection and septic shock.^[8]^ Thus, early prevention, diagnosis and treatment before, during, and after surgery are very important, and it is of great significance to the prognosis of patients. Therefore, we aimed to investigate the risk factors for acute urinary infection after retrograde upper urinary lithotripsy, to provide reliable evidence into the management of urolithiasis.

## 2 Methods

### 2.1 Ethics

Our study had been verified and approved by the ethical committee of medical research of Wuhan central hospital (190108), and written informed consents had been obtained from all the included patients.

### 2.2 Patients

Patients who received retrograde upper urinary lithotripsy from June 1, 2019 to December 31, 2020 were identified as potential population. The inclusion criteria were as follows: Adult patients with age ≥18 year; patients were diagnosed with kidney and ureteral calculi, and met the indications for retrograde lithotripsy and underwent retrograde upper urinary tract lithotripsy in our hospital. Exclusion criteria: patients with urinary tract infections before surgery; patients who have undergone lithotripsy treatment within the past 1 month; patients who are unwilling to participate in this study.

### 2.3 Retrograde upper urinary lithotripsy

The operation was conducted as follows: under anesthesia, the patient took the lithotomy position, used the F8∼9.8 rigid ureteroscope of MEDBIO company to enter the bladder under direct vision, to find the ureteral orifice, and entered the ureter by rotating the pick-up mirror. Then, the affected side of the ureter was explored to the renal pelvis. After confirming that the ureter was not accompanied by angle, twist, stenosis or atresia, a nickel-titanium guide wire was indwelled, up to the kidney, down to the bladder, and withdrawn from the hard scope. Then we placed the flexible ureteroscope expansion sheath along the nickel-titanium guide wire, entered the ureteral orifice to the renal pelvis, pulled out the inner core, exited the guide wire, and placed Storz fiber or electronic flexible ureteroscope along the outer sheath under direct vision to explore the renal pelvis and each calyx. After finding the stone, a 200 μm holmium laser fiber was inserted through the operating channel, and the holmium laser power was set to 10 to 15W. The stone was crushed by a continuous pulse method. After the lithotripsy was completed, one F5 double J tube and one F20 urinary tube were routinely placed. We did not give any prophylactic pre-op antibiotics in our study since the antibiotics are strictly controlled in our hospital and prophylactic pre-op antibiotics are not recommended in our practice.

Bladder irrigation was conducted to remove blood clots, bacteria in the urinary systems, to reduce wound exudation and reduce the infections. All patients were indwelled with F20 Foley three-chamber urinary catheter, and the balloon was injected with 50 to 60 mL of 0.9% saline solution. After the operation, the patient returned to the ward and was given 3 L 0.9% saline for bladder irrigation, and the temperature of solution was controlled at 30 to 35°C. The height of the irrigation fluid is about 70 cm above the bladder. We controlled the flushing speed at 80 to 120 drops per minute. We gradually reduced the washing speed according to the color change of the drained washing liquid.

### 2.4 The diagnosis of urinary tract infection

We monitored the urinary tract infection in the postoperative days, asymptomatic patients with positive urinary culture were considered as urinary tract infection according to the criterial of urinary tract infection guidelines,^[9,10]^ the diagnostic criteria for urinary tract infection in this study were: The patient's symptoms, medical history, and signs suggested urinary tract infection, with positive urine culture, or body temperature >38.5°C. The postoperative culture was taken start from postoperative day 1, and the urine specimen was routinely tested every 3 days after surgery. We excluded other system infections and other factors, once met the patients was diagnosed as urinary tract infection. All the infections including in patients who were asymptomatic were treated with Ceftriaxone 2 g/d for 3 days.

### 2.5 Data collection

The 2 authors independently collected relevant clinical data of included patients, including gender, age, body mass index, cases of diabetes, hypertension, hyperlipidemia, number of catheter intubations, stone diameters, duration of surgery, duration of urinary tube insertion and the treatment of bladder irrigation.

### 2.6 Statistical methods

SPSS 23.0 statistical software was adopted to analyze all data, and count data were expressed as the number of cases or percentages; the comparison of sample rates used Chi-Squared test. With the occurrence of urinary tract infection as the dependent variable, and the single factor analysis of the occurrence of urinary tract infection with meaningful influencing factors as independent variables, and the logistic regression model was used for the multivariate analysis. In this study, *P* < .05 indicated that the difference between the groups was statistically significant.

## 3 Results

### 3.1 The characteristics of included patients

A total of 410 patients received retrograde upper urinary lithotripsy in our hospital were included, of whom 62 patients had suffered from the urinary tract infection, the incidence of urinary tract infection in patients undergoing retrograde upper urinary lithotripsy was 15.12%. As presented in Table 1, there were significant differences in the gender, age, diabetes, stone diameter, duration of urinary tube insertion and duration of surgery between infection and no-infection group (all *P* < .05), and no significant differences in the body mass index, hypertension, hyperlipidemia and bladder irrigation between 2 groups.

**Table 1:** The characteristics of included patients.

Variables	Overall patients (n = 410)	Infection group (n = 62)	Non-infection group (n = 348)	χ^2^	*P*
Gender					
Male	231 (56.34%)	20 (32.26%)	211 (60.63%)	1.058	.008
Female	179 (43.66%)	42 (67.74%)	137 (39.37%)		
Age (y)					
<60	251 (61.22%)	24 (38.71%)	227 (65.23%)	1.202	.017
≥60	159 (38.78%)	38 (61.29%)	121 (34.77%)		
BMI (kg/m^2^)					
<25	295 (71.95%)	46 (74.19%)	249 (71.55%)	1.168	.108
≥25	115 (28.05%)	16 (25.81%)	99 (28.45%)		
Diabetes					
Yes	147 (35.85%)	36 (58.06%)	111 (31.90%)	1.317	.042
No	263 (61.15%)	26 (41.94%)	237 (68.10%)		
Hypertension					
Yes	190 (46.34%)	30 (48.39%)	160 (45.98%)	1.021	.096
No	220 (53.66%)	32 (51.61%)	188 (54.02%)		
Hyperlipidemia					
Yes	104 (25.37%)	19 (30.65%)	85 (24.43%)	1.195	.103
No	306 (74.63%)	43 (69.35%)	263 (75.57%)		
Stone diameter (cm)					
<2	277 (67.56%)	22 (35.48%)	255 (73.28%)	1.223	.014
≥2	133 (32.44%)	40 (64.52%)	93 (26.72%)		
Duration of urinary tube insertion (days)					
<3	236 (57.56%)	21 (33.87%)	215 (61.78%)	1.154	.022
≥3	174 (42.44%)	41 (66.13%)	133 (38.22%)		
Duration of surgery (min)					
<90	283 (69.02%)	24 (38.71%)	259 (74.43%)	1.123	.019
≥90	127 (30.98%)	38 (61.29%)	89 (25.57%)		
Bladder irrigation					
Yes	78 (19.02%)	11 (17.74%)	67 (19.25%)	1.413	.073
No	332 (80.98%)	51 (82.26%)	281 (80.75%)		

### 3.2 Pathogens distribution

A total of 62 pathogens were detected from the 62 patients with urinary tract infection. As showed in Table 2, the Escherichia coli (62.90%) was the most commonly seen bacterial in patients with urinary tract infection.

**Table 2:** Pathogens distribution of postoperative urinary tract infection (n = 62).

Pathogens	Number	Proportion (%)
Gram-positive bacteria	11	17.74
Staphylococcus aureus	5	8.06
Hemolytic Streptococcus	4	6.45
Staphylococcus epidermidis	2	3.23
Gram-negative bacteria	48	77.42
Escherichia coli	39	62.90
Pseudomonas aeruginosa	5	8.06
Enterobacter cloacae	2	3.23
Clostridium perfringens	2	3.23
Fungus	3	4.84
Candida albicans	3	4.84

### 3.3 Logistic regression analysis

The variable assignments of multivariate logistic regression were presented in Table 3. As showed in Table 4, female (odds ratio [OR]: 1.602, 95% confidence interval 95% [CI]: 1.132∼2.472), age > 50y (OR: 2.247, 95% CI: 1.346∼3.244), diabetes (OR: 2.228, 95%CI1.033∼3.451), stone diameter ≥2 cm (OR: 2.152, 95% CI: 1.395∼3.099), duration of urinary tube insertion ≥3 days (OR: 1.942, 95% CI: 1.158∼2.632), duration of surgery ≥90minutes (OR: 2.128, 95% CI: 1.104∼3.846) were the independent risk factors for the postoperative urinary tract infection in patients with retrograde upper urinary lithotripsy (all *P* < .05).

**Table 3:** The variable assignment of multivariate logistic regression.

Factors	Variables	Assignment
Infection	Y	yes = 1, no = 2
Gender	X_1_	Female = 1, male = 2
Age	X_2_	≥60 = 1, <60 = 2
Diabetes	X_3_	Yes = 1, No = 2
Stone diameter (cm)	X_4_	≥2 = 1, <1 = 2
Duration of urinary tube insertion (days)	X_5_	≥3 = 1, <3 = 2
Duration of surgery (min)	X_6_	≥90 = 1, <90 = 2

**Table 4:** Logistic regression analysis on the risk factors of postoperative urinary tract infection in patients with retrograde upper urinary lithotripsy.

Variables	β	S $\bar{\text{x}}$	OR	95% CI	*P*
Female	0.214	0.238	1.602	1.132∼2.472	.024
Age >50 y	0.871	0.112	2.247	1.346∼3.244	.032
Diabetes	0.405	0.124	2.228	1.033∼3.451	.019
Stone diameter ≥2 cm	0.834	0.215	2.152	1.395∼3.099	.025
Duration of urinary tube insertion ≥3 d	0.634	0.243	1.942	1.158∼2.632	.048
Duration of surgery ≥90 min	0.779	0.191	2.128	1.104∼3.846	.017

## 4 Discussions

The incidence of urinary tract stones remains high. The incidence of stone in population worldwide is about 1% to 5%, among which upper urinary tract stones account for about 70%.^[11,12]^ With the development of minimally invasive techniques, intracavitary lithotripsy for upper urinary tract stones has now become a first-line surgical method for upper urinary tract stones.^[13]^ Compared with traditional open surgery, intracavitary lithotripsy for upper urinary tract stones has the advantages of less trauma, faster recovery and fewer complications.^[14–16]^ However, urinary infections and a delay in the diagnosis and treatment of ureteral obstruction represents the most important prognostic factor for worse results in terms of renal function recovery.^[17]^ urinary tract infection is the main postoperative complication, and severe cases can cause systemic inflammation. The clinical manifestations of response syndrome may even progress to septic shock or multiple organ dysfunction, and even death of the patient.^[18]^ Therefore, to clarify the related risk factors and mechanism of urinary tract infection after retrograde upper urinary lithotripsy is very important for the prognosis of patients with retrograde upper urinary lithotripsy. The results of this present study have revealed that the incidence of urinary tract infection in patients undergoing retrograde upper urinary lithotripsy was 15.12%, Escherichia coli was the most commonly-seen bacteria, and female, age >50 years, diabetes, stone diameter ≥2 cm, duration of urinary tube insertion ≥3 days, duration of surgery ≥90 minutes were the independent risk factors for the postoperative urinary tract infection in patients with retrograde upper urinary lithotripsy, corresponding measures are needed on those risk factors to prevent the treatment of postoperative urinary tract infection in patients with retrograde upper urinary lithotripsy.

Female patients are more likely to develop urinary tract infections after upper urinary tract lithotripsy than male patients.^[19]^ This may be due to the greater risk of urinary tract infections in female patients than male patients. It may also be related to factors such as the difficulty of maintaining the hygiene of the female perineum and the senile atrophic vaginitis caused by the fall of estrogen level during menopause.^[20,21]^ However, it may not explain the work in acute setting of ureteroscopy with regards to difference population and treatment practice. Patients with age ≥60 years are more likely to have urinary tract infection. The mechanism may be related to the poorer immune function and body response of the elderly.^[22]^ The immune function of diabetes patients is generally low, easily leading to infection. The mechanism may be that hyperglycemia increases the plasma osmotic pressure and inhibits the function of immune cells.^[23,24]^ The existence of metabolic disorders also leads to the weakening of the function of phagocytosis and sterilization of white blood cells, and the acute and chronic complications caused by diabetes can further aggravate the damage of immune function.^[25–27]^ In addition, the high sugar content in the urine of diabetic patients is easy for strain growth.^[28]^

Stone size is related to urinary tract infection, larger stones contain more bacteria and toxins, and that larger stones require longer operation time.^[29]^ At the same time, larger stones are more likely to cause obstruction and lead to infection.^[30]^ Some studies^[31,32]^ have found that the risk of urinary tract infection after intracavitary lithotripsy of upper urinary tract stones will increase significantly when the operation time exceeds the critical value of 102 minutes through the receiver operating characteristic curve (ROC). When the operation time of the only known variable is prolonged, the absorption of the perfusate will correspondingly increase, thereby increasing the patient's risk of urinary tract infection and endotoxin.^[33]^ It is suggested that the surgeon should try to control the duration of surgery within 90 minutes.

Early prevention and intervention of urinary tract infection is very important. Routine urine examinations should be performed before surgery, urine culture and drug sensitivity should be added, sensitive antibiotics should be actively used, and changes in urine white blood cells should be dynamically monitored.^[34,35]^ Strictly abide by the principles of aseptic operation during the operation, improve lithotripsy skills, reduce the number of lens visits, and reduce damage to the urinary tract.^[36]^ At the same time, the operation time should be strictly controlled.^[37]^ Actively control the patient's blood glucose level during the perioperative period, which is conducive to the recovery of patients after intracavitary lithotripsy and reduces the occurrence of urinary tract infections.^[38,39]^

Several limitations in this present study must be concerned. Firstly, the sample size in this study was relatively small, it might be underpowered to detect the differences between groups and potential risks, future studies with larger sample size and different areas are needed. Secondly, in particular a study^[40]^ showed that patients who experience delayed relief of ureteral obstruction had decreased long-term renal function as suggested by the lower values of estimated creatinine clearance and mercaptoacetyltriglycine clearance, and were at risk for hypertension or exacerbation of preexisting hypertension, due to limited data, we could not include it for analyses. Besides, catheter duration more than 3 days is not commonly seen in clinical practice, 174 (42.44%) patients in our study had catheter duration more than 3 days, which may be related to different region and hospital practice, therefore, the results should be treated with cautions. In the future, more prospective studies with larger sample size are warranted to further elucidate the potential risks of risk factors of urinary infection in patients with retrograde upper urinary lithotripsy, to provide reliable evidences to the clinical practice and treatment.

## 5 Conclusions

At present, retrograde upper urinary lithotripsy has become the main treatment for upper urinary tract stones. Therefore, it is important to understand the risk factors of urinary infection after intracavitary lithotripsy to effectively reduce and prevent the occurrence of postoperative urinary tract infections. The results of this study show that female, age >50 year, diabetes, stone diameter ≥2 cm, duration of urinary tube insertion ≥3 days, duration of surgery ≥90 minutes are the independent risk factors for urinary infection after lithotripsy. Therefore, for the patients with above-mentioned factors, we should be vigilant after surgery to avoid the occurrence and progression of urinary infection. Future research should continue to screen and analyze the risk factors related to urinary tract infection after upper urinary tract lithotripsy, so as to better guide the early warning, diagnosis and treatment of postoperative urinary tract infection.

## Author contributions

**Conceptualization:** Chuan Peng.

**Data curation:** Jun Xu.

**Investigation:** Jun Xu, Zhaozhao Chen, Chuan Peng.

**Methodology:** Chuan Peng.

**Project administration:** Zhaozhao Chen.

**Resources:** Jun Xu, Zhaozhao Chen.

**Software:** Jun Xu.

**Supervision:** Zhaozhao Chen.

**Validation:** Zhaozhao Chen, Chuan Peng.

**Visualization:** Zhaozhao Chen.

**Writing – original draft:** Zhaozhao Chen.
